# Complete genome sequence of *Bacillus subtilis* subsp. *natto* NARUSE using PacBio sequencing

**DOI:** 10.1128/MRA.00578-23

**Published:** 2023-11-20

**Authors:** Yukie Kubo, Yoshiyuki Tanizaki, Daisuke Hira, Yasumune Nakayama, Chihiro Kadooka, Takuji Oka

**Affiliations:** 1 Kyushu Soy Food Co., Ltd., Kumamoto, Japan; 2 Marukin Foods Co., Ltd., Kumamoto, Japan; 3 Department of Biotechnology and Life Sciences, Faculty of Biotechnology and Life Sciences, Sojo University, Kumamoto, Japan; 4 Division of Applied Microbial Technology, Graduate School of Engineering, Sojo University, Kumamoto, Japan; University of Delaware College of Engineering, Newark, Delaware, USA

**Keywords:** *Bacillus subtilis*, natto, genome, PacBio

## Abstract

We report the complete genome sequence of *Bacillus subtilis* subsp. *natto* NARUSE, which has been traditionally employed for fermenting soybeans in Japan. The genome was sequenced using the PacBio system, yielding a sequence, yielding a sequence length of 4,148,793 nucleotides for the circular chromosome and 62,770 nucleotides for the plasmid.

## ANNOUNCEMENT

*Bacillus subtilis* subsp. *natto* NARUSE is an industrially bacterial strain utilized in producing a traditional fermented soybean food known as “*natto*” in Japan. Different strains of *B. subtilis* subsp. *natto*, such as MIYAGINO (also known as BEST195), NARUSE, and TAKAHASHI, are used for *natto* production (1). The complete genome sequence in BEST195 strain has been revealed by combining long and short reads (2). Although draft genomes of the NARUSE and TAKAHASHI strains have been published (1), the complete genome sequence of NARUSE strain has not yet been reported. In this study, we employed a PacBio Sequel lle sequencer to obtain the complete genome and plasmid sequences of NARUSE strain.

*B. subtilis* subsp. *natto* NARUSE was purchased from the Naruse Fermentation Chemical Laboratory K.K. (Tokyo, Japan). The cells were grown in a liquid medium containing 1% glucose, 0.2% (NH_4_)_2_SO4, 0.4% KH_2_PO_4_, 0.1% K_2_HPO_4_, 0.025% MgSO_4_·7H_2_O, 10µg/ml of biotin, and all amino acids [0.02% (wt/vol) each] at 37°C for 24 h. Genomic DNA was extracted using the NucleoBond HMW DNA Kit (Macherey-Nagel, Düren, Germany) and purified using the DNeasy PowerClean Pro Cleanup Kit (QIAGEN, Venlo, Netherlands), and short DNA fragments were removed using the Short Read Eliminator XS Kit (PacBio, CA, USA). The DNA fragments were fragmented using g-TUBE (Covaris, MA, USA) to approximately 10–20 kbp. A library was constructed using the SMRTbell template prep kit v2.0 (PacBio). The polymerase complexes within the libraries were formed using Binding Kit 2.2 (PacBio). These complexes were subjected to sequencing using the Sequel IIe system. The adapter overhang sequence was eliminated from the sequenced data to generate subreads using SMRT Link (v.11.0.0.146107) with the default parameters. High-fidelity (HiFi) reads with quality values greater than 20 or exhibiting an accuracy of 99% were acquired by the SMRT Link. HiFi reads below 1,000bp were removed using Filtlong (v.0.2.1). HiFi reads exceeding 1,000bp were *de novo* assembled using Flye (v.2.9.1-b1780) with the default parameters (3). The genome coverage was 58.6×, and the N50 value was 4,148,793 bp. The integrity of the assembled genome data was assessed using CheckM (ver. 1.2.2) and the *Bacillus*-specific marker sets, which showed a 99.8% integrity rate (4). We obtained two circular contigs represented by a complete chromosome measuring 4,148,793bp and a plasmid measuring 62,770bp. The plasmid has been designated as pNARUSE. The pNARUSE sequence exhibits a pronounced resemblance to that of pLS20 as discerned through BLAST (Basic Local Alignment Search Tool) analysis (5, 6). Annotation was performed with DFAST v1.2.18 (7) using MetaGeneAnnotator v2008/08/19 (8) for coding sequence, Barrnap v0.8 (9) for rRNA, and ARAGORN v1.2.38 (10) for tRNA. Default parameters were used for all software. Our analysis predicted 4,422 protein-encoding genes, 88 tRNA genes, and 30 rRNA genes (10 of 5S-rRNA, 10 of 16S-rRNA, and 10 of 23S-rRNA).

## Data Availability

The complete sequences of NARUSE genome and pNARUSE have been deposited in NCBI/ENA/DDBJ under accession numbers AP028059 and AP028060. The sequencing data can be found in the DDBJ Sequence Read Archive under the accession number DRX447375.
